# Supplementary material for: Determinants of Changes in Women’s and Men’s Physical Activity and Sedentary Behavior across the Transition to Parenthood: A Focus Group Study
Source: Int J Environ Res Public Health. 2022 Feb 19;19(4):2421. doi: 10.3390/ijerph19042421 (PMC8878758; doi:10.3390/ijerph19042421)
Supplement: Supplementary file 1 [file ijerph-19-02421-s001.zip › ijerph-1582109-supplementary.pdf]

Table S1: Categorization (leaf-category, stem-category and main level) of determinants of changes in PA behavior during pregnancy and postpartum

Determinant	Leaf-category	Stem-category	Main level	Determinant	Leaf-category	Stem-category	Main level
**DURING PREGNANCY**				**POSTPARTUM**			
**Habits**	PA habits	Psychological	Individual				
**Health consciousness**	Health cognitions	Psychological	Individual				
Fit for delivery motivation	Health cognitions	Psychological	Individual				
Healthiness	Health cognitions	Psychological	Individual				
Wellbeing	Health cognitions	Psychological	Individual				
**Mood and emotions**	Mood and emotions	Psychological	Individual				
Body image	Weight control cognitions and behaviors	Psychological	Individual				
Mental tiredness	Mood and emotions	Psychological	Individual				
Stress	Mood and emotions	Psychological	Individual				
**PA knowledge**	PA knowledge, skills and abilities	Psychological	Individual	**PA knowledge**	PA knowledge, skills and abilities	Psychological	Individual
				**Parenthood perceptions and responsibilities**	Personality	Psychological	Individual
				Barriers to ask for help	Personality	Psychological	Individual
				Barriers to leave child	Personality	Psychological	Individual
				Barriers to self-care	Personality	Psychological	Individual
				Worries and uncertainties about care for baby	Personality	Psychological	Individual
**Self-licensing (pregnancy card)**	Self-regulation	Psychological	Individual	**Self-licensing**	Self-regulation	Psychological	Individual
**Self-regulation**	Self-regulation	Psychological	Individual	**Self-regulation**	Self-regulation	Psychological	Individual
Self-control	Self-regulation	Psychological	Individual	Habituality of physical activity	PA habits	Psychological	Individual
Self-efficacy	Personality	Psychological	Individual	Planning	Self-regulation	Psychological	Individual
Self-regulation	Self-regulation	Psychological	Individual	Self-control	Self-regulation	Psychological	Individual
Weight control	Weight control cognitions and behaviors	Psychological	Individual	Self-regulation	Self-regulation	Psychological	Individual

				Weight control	Weight control cognitions and behaviors	Psychological	Individual
**Worries and safety concerns**	Worries and concerns	Psychological	Individual	**Worries and safety concerns**	Worries and concerns	Psychological	Individual
**Other priorities**	Situational and time constraints	Situational	Individual	**Other priorities**	Situational and time constraints	Situational	Individual
**Practical and situational constraints**	Situational and time constraints	Situational	Individual	**Practical and situational constraints**	Situational and time constraints	Situational	Individual
No suitable sport clothes	Situational and time constraints	Situational	Individual	Practical issues	Situational and time constraints	Situational	Individual
Other situational constraints	Situational and time constraints	Situational	Individual	Organizational issues	Situational and time constraints	Situational	Individual
**Time constraints and opportunities**	Situational and time constraints	Situational	Individual	**Time constraints and opportunities**	Situational and time constraints	Situational	Individual
Time constraints	Situational and time constraints	Situational	Individual				
Time opportunities	Time opportunities	Situational	Individual				
**Physiology**	Physiology	Biological	Individual	**Physiology and physical health**	Physiology	Biological	Individual
Discomfort	Physiology	Biological	Individual	Recovery after pregnancy/delivery	Physiology	Biological	Individual
Fatigue	Physiology	Biological	Individual	Fatigue	Physiology	Biological	Individual
Physiological changes	Physiology	Biological	Individual	Physiological changes	Physiology	Biological	Individual
(Perceived) physical restrictions	Physiology	Biological	Individual				
**Social influence**	Social influence	Social	Interpersonal	**Social influence**	Social influence	Social	Interpersonal
Advice	Social influence	Social	Interpersonal	Influence of male partner	Social influence	Social	Interpersonal
Lack of other people’s knowledge	Social influence	Social	Interpersonal				
Imposed physical restrictions	Social influence	Social	Interpersonal	Influence of female partner	Social influence	Social	Interpersonal
Influence of female partner	Social influence	Social	Interpersonal	Professional influence	Social influence	Social	Interpersonal
Professional influence	Social influence	Social	Interpersonal	Social activities	Social influence	Social	Interpersonal
Social discouragement	Social influence	Social	Interpersonal				
Social support	Social influence	Social	Interpersonal				
				**Influence of baby**	Social influence	Social	Interpersonal
				Adaptation to the rhythm of baby	Social influence	Social	Interpersonal
				Baby becomes priority	Social influence	Social	Interpersonal

				Care for baby	Social influence	Social	Interpersonal
				Other activities because of baby	Social influence	Social	Interpersonal
				Practical organization around baby	Social influence	Social	Interpersonal
				Practical constraints because of baby	Social influence	Social	Interpersonal
				Role model	Social influence	Social	Interpersonal
**Environment PA opportunities**	Environment PA availability and accessibility	Meso/Macro	Environment				
**Product price**	Market prices	Meso/Macro	Environment	**Product price**	Market prices	Meso/Macro	Environment
**Pregnancy leave regulations**	Governmental regulations	Government	Policy	**Maternity leave regulations**	Governmental regulations	Government	Policy

PA: Physical activity

Table S2: Categorization (leaf-category, stem-category and main level) of determinants of changes in SB during pregnancy and postpartum

Determinant	Leaf-category	Stem-category	Main level	Determinant	Leaf-category	Stem-category	Main level
**DURING PREGNANCY**				**POSTPARTUM**			
**Self-licensing (pregnancy card)**	Self-regulation	Psychological	Individual				
**Self-regulation**	Self-regulation	Psychological	Individual	**Self-regulation**	Self-regulation	Psychological	Individual
				Planning	Self-regulation	Psychological	Individual
**Change in activities**	Situational and time constraints	Situational	Individual	**Change in activities**	Situational and time constraints	Situational	Individual
Fewer out-of-home activities	Situational and time constraints	Situational	Individual	Fewer out-of-home activities	Situational and time constraints	Situational	Individual
Preparing child room	Situational and time constraints	Situational	Individual	More household activities	Situational and time constraints	Situational	Individual
				**Time constraints**	Situational and time constraints	Situational	Individual
**Work situational**	Situational and time constraints	Situational	Individual	**Work situational**	Situational and time constraints	Situational	Individual
**Physiology and physical health**	Physiology	Biological	Individual	**Physical health**	Physiology	Biological	Individual
Discomfort	Physiology	Biological	Individual				
Fatigue	Physiology	Biological	Individual				
**Social influence**	Social influence	Social	Interpersonal				
Support of male partner	Social influence	Social	Interpersonal				
Influence of female partner	Social influence	Social	Interpersonal				
Professional support	Social influence	Social	Interpersonal				
External support	Social influence	Social	Interpersonal				
				**Influence of baby**	Social influence	Social	Interpersonal
				Adaptation to the rhythm of baby	Social influence	Social	Interpersonal
				Care for baby	Social influence	Social	Interpersonal
				Practical organization around baby	Social influence	Social	Interpersonal

				Feeding baby	Social influence	Social	Interpersonal
**Pregnancy leave regulations**	Governmental regulations	Government	Policy	**Maternity leave regulations**	Governmental regulations	Government	Policy
